# Comparing Ultrasound- and Laparoscopic-Guided Transversus Abdominis Plane (TAP) Blocks in Sleeve Gastrectomy Patients

**DOI:** 10.7759/cureus.76033

**Published:** 2024-12-19

**Authors:** William C Baumgartner, Fielding M Richards, Brandon J Arnsberger, Deirdre Sheets, John Powell

**Affiliations:** 1 General Surgery, University of Pittsburgh Medical Center (UPMC) Community Osteopathic Hospital, Harrisburg, USA; 2 General and Bariatric Surgery, University of Pittsburgh Medical Center (UPMC) Community Osteopathic Hospital, Harrisburg, USA

**Keywords:** bariatric surgery, laparoscopic transversus abdominis plane block, local anesthesia, opioid use, postoperative pain, regional anesthesia, transversus abdominis plane block

## Abstract

Introduction

Obesity is a major disease process in the United States with increasing prevalence and is associated with various comorbid conditions. Bariatric surgery, particularly laparoscopic sleeve gastrectomy (LSG), is an effective weight loss intervention but presents challenges in postoperative pain management. This study compares the effectiveness of ultrasound-guided transversus abdominis plane (UTAP) blocks, laparoscopic-guided transversus abdominis plane (LTAP) blocks, and no regional anesthesia on overall opioid use and postoperative outcomes in LSG patients.

Methods

This retrospective cohort study included 1,239 obese patients who underwent LSG at a single hospital from January 2019 to June 2023. Patients were categorized into the following three groups: UTAP, LTAP, and no TAP. The primary outcome was the total morphine milligram equivalents (MME) used within the first 24 h and 48 h, and the average amount of MME during the hospital stay (all reported in median interquartile ranges). Secondary outcomes included total anesthesia time, postoperative pain scores, length of stay (LOS), 30-day readmission, and emergency room visit rates. Statistical analyses included chi-square tests, Fisher’s exact test, independent t-tests, Mann-Whitney U tests, and multiple linear regression.

Results

The UTAP group had significantly lower median MME within the first 24 h (63 MME) compared to the LTAP group (98.8 MME, p<0.0001) and throughout the hospital stay (93 MME vs. 120 MME, p=0.0004). Both UTAP and LTAP groups had significantly lower total MME compared to the no TAP group (p<0.0001 for both). Total anesthesia time was longer for UTAP (98.5 min) compared to LTAP (92.5 min, p=0.0472). LOS was significantly longer in the UTAP group (1.59 days) compared to LTAP (1.18 days, p=0.0061). There were no significant differences in pain scores at various time points or in 30-day readmission and emergency room visit rates between the UTAP and LTAP groups.

Conclusion

UTAP blocks demonstrated reduced opioid use compared to LTAP and no TAP, likely due to enhanced accuracy with ultrasound guidance. Further prospective studies are warranted to confirm these findings and refine pain management strategies for bariatric surgery patients.

## Introduction

Obesity as a disease, defined as a body mass index (BMI) >30, and severe obesity, defined as a BMI >40, is associated with a number of serious comorbid conditions [1]. According to the Centers for Disease Control and Prevention, the age-adjusted prevalence of obesity in the United States increased from 30.5% in 1999-2000 to 42.4% in 2017-2018. Similarly, the age-adjusted prevalence of severe obesity increased from 4.7% to 9.2% during this period [2]. Bariatric surgery has emerged as a popular and effective means of achieving weight loss in individuals who failed to do so with lifestyle modification alone. Patients may lose as much as 60% of excess weight six months after surgery, and 77% of excess weight as early as 12 months after surgery [3].

Postoperative pain control following bariatric surgery can be challenging despite the use of laparoscopic techniques. This difficulty arises from the number of trocar sites needed for adequate intraoperative visualization and the increased subcutaneous fat deposition in patients with a higher BMI [4]. Additionally, it is well known that morbidly obese patients present a unique challenge to perioperative care due to associated cardiovascular and respiratory comorbidities [5]. Obstructive sleep apnea (OSA) is a common comorbidity associated with obesity that increases the likelihood of postoperative complications [6]. Opioid use has been linked to central apnea and can exacerbate obstructive apnea in obese patients by suppressing respiratory drive [7]. Additionally, higher doses of opioids are associated with prolonged time until the return of bowel function, decreased ambulation, prolonged hospital stay, and increased risk of venous thromboembolisms (VTEs) [6].

Multimodal pain management involves using two or more non-opioid medications that act through different pathways to achieve analgesia while reducing opioid consumption and related side effects [8]. Transversus abdominis plane (TAP) blocks are part of this multimodal approach for abdominal surgeries, providing analgesic benefits and reducing postoperative opioid requirements [9]. Prior studies have shown that TAP blocks are associated with decreased postoperative opioid use and are superior to local anesthetic port site infiltration alone [10,11]. TAP blocks also improved outcomes such as reduced nausea and shorter hospital stays compared to intravenous postoperative analgesics alone [12].

Ideally, a TAP block is performed under ultrasound guidance to ensure accurate targeting of the transversus abdominis plane; however, TAP blocks can also be performed under laparoscopic visualization [13]. Zaghiyan et al. hypothesized that laparoscopic-guided TAP blocks (LTAP) are non-inferior to ultrasound-guided TAP blocks (UTAP) in a randomized clinical trial comparing LTAP and UTAP in minimally invasive colorectal surgery. The authors reported that LTAP was superior to UTAP in achieving pain control and minimizing opioid use in the first 24 h after colorectal surgery [14].

However, current literature comparing UTAP and LTAP in bariatric surgery is limited, and no studies have definitively assessed which method is more effective for managing postoperative pain in this specific patient population. Given the unique challenges posed by morbid obesity - particularly the increased abdominal wall thickness, which may affect the efficacy of TAP blocks - there is a clear need to assess the comparative effectiveness of these techniques in bariatric patients. This study addresses this gap by directly comparing UTAP and LTAP in laparoscopic sleeve gastrectomy (LSG) patients, hypothesizing that UTAP’s precision in local anesthetic placement due to ultrasound guidance may lead to improved outcomes in bariatric patients.

The TAP block is a regional anesthesia technique that involves injecting a local anesthetic into the lateral abdominal wall plane between the internal oblique and transversus abdominis muscles, which contain the T6-L1 thoracolumbar nerves [9]. This retrospective study aimed to compare the effects of UTAP vs. LTAP and evaluate their overall effectiveness compared to no regional anesthesia. We hypothesize that UTAP is more effective due to the accuracy of ultrasound-guided placement within the correct plane.

## Materials and methods

Study design

Prior to data collection, the study received approval from the University of Pittsburgh Medical Center (UPMC) Institutional Review Board (#23E031). This retrospective cohort study included data on all patients diagnosed with morbid obesity who underwent laparoscopic sleeve gastrectomy (LSG) by four bariatric surgeons between January 2019 and June 2023 at a MBSAQIP Certified high volume bariatric program at a single community hospital in central Pennsylvania. No patient-identifiable information was used and it was considered a low-risk study; patient consent was not obtained. Patients who underwent open surgical procedures were excluded. Multiple anesthesia providers participated in the care of LSG patients during the study period. All LTAPs and UTAPs were administered at the discretion of the supervising surgeon and anesthesiologist, with UTAPs administered under the supervision of an attending anesthesiologist. The study included patients aged 19-81 years with American Society of Anesthesiologists (ASA) physical status classification II-IV.

Interventions

Patients received standard intraoperative and postoperative pain control using narcotics as well as non-narcotics according to the institutional LSG protocol. Narcotics used included fentanyl, morphine, hydromorphone, oxycodone, hydrocodone, and tramadol. These medications were administered as needed based on the nursing pain scale protocol. No patients received scheduled narcotic dosing. Non-narcotic pain control options included acetaminophen, either alone or in combination with oxycodone or hydrocodone formulations. The standard dose of acetaminophen was 1,000 mg every 8 h, unless the patient had significant hepatic dysfunction. All patients received scheduled acetaminophen dosing.

Outcomes

The primary outcomes of this study were to assess the total amount of morphine milligram equivalents (MME) within the first 24 h, 48 h, and the average amount of MME during the hospital stay (all reported in median interquartile ranges). The two-day endpoint was chosen as most patients were discharged within one to two days. Secondary outcomes included total anesthesia time, postoperative pain scores, length of stay (LOS), 30-day readmission rate, and 30-day emergency room visit rate. Postoperative pain scores were recorded upon arrival in the postanesthesia care unit (PACU), 1 h after arriving in the PACU, and on postoperative day one. Total anesthesia time was defined as the period from initial anesthesia administration to discontinuation. TAP blocks were performed while the patient was under anesthesia. Patient demographics, including age, gender, BMI, and ASA rating were also collected.

Surgery

LSG was performed under general endotracheal anesthesia, with intraoperative monitoring including end-tidal CO_2_, arterial saturation, blood pressure, electrocardiography, and temperature. The method of TAP administration was decided by the anesthesiologist and surgeon prior to the induction of anesthesia. Surgery was performed using a standardized stepwise manner with a six-port technique, with some variability in port placement at the discretion of the attending surgeon. If an LTAP block was performed, it was done at the beginning of the case.

UTAP block technique

Informed consent was obtained for all UTAP blocks. Following induction of anesthesia and intubation, the abdomen was prepped sterilely. An ultrasound probe was used to identify the lateral interfascial plane between the transversus abdominis (TA) and internal oblique (IO) muscles. The probe was placed in a transverse plane along the lateral abdominal wall, starting at the midaxillary line and adjusted for optimal view. The interfascial planes, including the peritoneal lining, transversus abdominis, internal oblique muscle, external oblique muscle, and soft tissues, were identified. A 4-inch, 21-gauge block needle was inserted under direct visualization into the space between the TA muscle and IO muscle. After confirming appropriate placement with 1-2 mL of local anesthetic mixture, 30 mL of plain 0.5% ropivacaine was injected under continuous ultrasound visualization. The anesthesia protocol for all UTAP blocks specified the use of 0.5% ropivacaine, with no bupivacaine used in this group. The procedure was then performed as described above on the opposite side.

Laparoscopic TAP block

In the LTAP group, after induction of general anesthesia, the abdomen was prepped and draped in a sterile fashion. A laparoscope was introduced, and an area on the abdominal wall between the lower costal margins and the iliac crest was identified. A 22 gauge 3.5-inch spinal needle was inserted through the skin and advanced under laparoscopic visualization. As the needle passed through the external and internal oblique fascia, two distinct "pops" were felt. If the needle traveled beyond the transversus abdominis muscle, it was slowly withdrawn until proper placement was confirmed. Bupivacaine 0.25% solution was initially injected to visualize hydrodissection. Once correct placement was confirmed, 30 mL of bupivacaine was injected into both the left and right lateral abdominal walls for a total of 60 mL. The procedure was then performed as described above on the opposite side. Patients in the no TAP group only received 30 mL of 0.25% bupivacaine with epinephrine around port sites at the end of surgery.

Statistical analysis

Statistical analysis was performed by our institutional statistician. Categorical variables were summarized as number and percent, and study groups were compared using the chi-square test or Fisher's exact test. Continuous variables were summarized as mean±SD and range if data were normally distributed. Non-normally distributed data were summarized as median and interquartile ranges. The comparisons were analyzed using an independent t-test or a Mann-Whitney U test, as appropriate. Multiple linear regressions were used to assess the relationship between MME and predictors for narcotic use. Regression coefficients and p-values were reported. A p-value less than 0.05 is considered statistically significant. All the analyses were done in SAS version 9.4 (Cary, NC: SAS Institute).

## Results

This trial included 1,239 patients who underwent LSG between January 2019 and June 2023 at a single community hospital in central Pennsylvania. Table 1 provides a detailed comparison of patient demographics across the following three groups: UTAP (n=88), LTAP (n=172), and no TAP (n=979). The mean age in the UTAP group was 57.7 years (SD: 12.3, range: 32-81 years), significantly older compared to the LTAP group (mean: 47.5 years, SD: 12.4, range: 19-76 years) and no TAP group (mean: 47.1 years, SD: 11.9, range: 20-77 years) (p=0.001). Most of the patients were female with 72.73% in UTAP, 79.65% in LTAP, and 80.29% in the no TAP group. Gender distribution was similar across groups (p>0.05). The mean BMI for the UTAP group was 47.0 (SD: 7.7, range: 35.8-78.2), for the LTAP group it was 46.2 (SD: 7.3, range: 30.4-77.0), and for the no TAP group it was 46.7 (SD: 7.4, range: 31.1-80.4). The mean BMI was not significantly different between groups (p>0.05). There were significant differences in ASA classification among the groups. In the UTAP group, 59.09% were ASA IV as compared to 79.65% in the LTAP group and 81.00% in the no TAP group who were ASA III (p<0.0001) (Table 1).

**Table 1:** Clinical characteristics and demographics of patients undergoing UTAP, LTAP, and no TAP. Data are presented as mean±SD and range for continuous variables (age and BMI) and as number and percentage for categorical variables (gender and ASA physical status rating). The American Society of Anesthesiologists Physical Status Classification System categorizes patients as ASA IV for incapacitating conditions, ASA III for severe systemic conditions, and ASA II for mild systemic conditions. P-values indicate statistical comparisons across groups (UTAP vs. LTAP, UTAP vs. no TAP, and LTAP vs. no TAP), with values <0.05 considered statistically significant. UTAP: ultrasound-guided transversus abdominis plane block; LTAP: laparoscopic-guided transversus abdominis plane block; BMI: body mass index (kg/m²); n: total number of patient; ASA: American Society of Anesthesiologists

Patient demographics	Ultrasound TAP (n=88)		Laparoscopic TAP (n=172)		No TAP (n=979)		p-Value		
	Mean (SD)	Range	Mean (SD)	Range	Mean (SD)	Range	UTAP vs. LTAP	UTAP vs. no TAP	LTAP vs. no TAP
Age (years)	57.7 (12.3)	32-81	47.5 (12.4)	19-76	47.1 (11.9)	20-77	<0.0001	<0.0001	0.6999
BMI	47.0 (7.7)	35.8-78.2	46.2 (7.3)	30.4-77.0	46.7 (7.4)	31.1-80.4	0.4319	0.7636	0.3950
Gender	Number	Percentage	Number	Percentage	Number	Percentage	0.2072	0.0955	0.8669
Male	24	27.27%	35	20.35%	194	19.82%			
Female	64	72.73%	137	79.65%	786	80.29%			
ASA rating	Number	Percentage	Number	Percentage	Number	Percentage	<0.0001	<0.0001	0.0191
Incapacitating (ASA IV)	52	59.09%	28	16.28%	100	10.21%			
Severe systemic (ASA III)	34	38.64%	137	79.65%	793	81.00%			
Mild systemic (ASA II)	2	2.27%	7	4.07%	86	8.78%			

Looking at primary outcomes for patients who received UTAP vs. LTAP, the median total amount of MME within the first 24 h was significantly lower in the UTAP group at 63 MME (interquartile range {IQR}: 30-95) compared to 98.8 MME (IQR: 75-130) in the LTAP group (p<0.0001). On the second day, the median MME was similar between the groups, with UTAP at 30 MME (IQR: 15-45) and LTAP at 23 MME (IQR: 15-45) (p=0.6595). The total MME during the entire stay was significantly lower in the UTAP group, with a median of 93 MME (IQR: 45-140) compared to 120 MME (interquartile range: 84-165) in the LTAP group (p=0.0004) (Table 2).

**Table 2:** Comparison of primary and secondary outcomes of UTAP blocks vs. LTAP blocks in postoperative pain management. Primary outcomes include total MME administered during the first 24 h, second postoperative day, and entire hospital stay, presented as the median and IQR. Secondary outcomes include total anesthesia time (median, IQR), pain scores (based on a pain scale ranging from 0 {no pain} to 10 {worst possible pain}, used to assess patients' subjective pain levels) at various postoperative time points (mean±SD, and range), length of hospital stay (mean±SD, range), and incidence of 30-day readmission and emergency room visits (number, percentage). P-values indicate statistical comparisons between UTAP and LTAP groups, with significance considered at p<0.05. UTAP: ultrasound-guided transversus abdominis plane block; LTAP: laparoscopic-guided transversus abdominis plane block; MME: morphine milligram equivalents; PACU: postanesthesia care unit; SD: standard deviation; IQR: interquartile range

Primary outcomes	Ultrasound TAP		Laparoscopic TAP		p-Value
	Median	Interquartile range	Median	Interquartile range	
Total amount of milligrams of morphine equivalents (MME) within the first 24 h	63	30-95	98.8	75-130	<0.0001
Total amount of milligrams of morphine equivalents (MME) 2nd day	30	15-45	23	15-45	0.6595
Total amount of milligrams of morphine equivalents (MME) during the entire stay	93	45-140	120	84-165	0.0004
Secondary outcomes					
Total anesthesia time	98.5	86-117	92.5	83-104.5	0.0472
	Pain score mean (SD)	Pain score range	Pain score mean (SD)	Pain score range	
Pain score entering PACU	2.48 (3.7)	0-10	2.35 (3.4)	0-10	0.7818
Pain score 1 h into PACU	5.31 (3.4)	0-10	5.44 (2.5)	0-10	0.7554
Pain score postoperative day 1	4.07 (2.5)	0-10	4.52 (2.7)	0-10	0.2096
Pain score at discharge	3.94 (2.7)	0-10	4.05 (2.1)	0-9	0.7408
Length of stay	Mean (SD)	Range	Mean (SD)	Range	0.0061
	1.59 (1.3)	0-10	1.18 (0.8)	0-5	
Readmission rates	Number	Percentage	Number	Percentage	p-Value
30-day readmission	3	3.41%	3	1.74%	0.4099
30-day emergency room visit	6	6.82%	13	7.56%	0.8283

When comparing secondary outcomes of UTAP with LTAP, total anesthesia time was slightly longer in the UTAP group, with a median of 98.5 min (IQR: 86-117) compared to 92.5 min (IQR: 83-104.5) in the LTAP group (p=0.0472). There were no significant differences in pain scores when entering PACU, 1 h in PACU, postoperative day (POD) 1, and discharge. Pain scores upon entering PACU were similar, with a mean of 2.48 (SD: 3.7, range: 0-10) in the UTAP group and 2.35 (SD: 3.4, range: 0-10) in the LTAP group (p=0.7818). One hour into PACU, the mean pain score was 5.31 (SD: 3.4, range: 0-10) for UTAP and 5.44 (SD: 2.5, range: 0-10) for LTAP (p=0.7554). On POD 1, pain scores were slightly lower in the UTAP group mean of 4.07 (SD: 2.5, range: 0-10) compared to the LTAP group mean of 4.52 (SD: 2.7, range: 0-10) (p=0.2096). At discharge, the mean pain score was 3.94 (SD: 2.7, range: 0-10) for UTAP and 4.05 (SD: 2.1, range: 0-9) for LTAP (p=0.7408). The length of stay (LOS) was significantly longer in the UTAP group, with a mean of 1.59 days compared to 1.18 days in the LTAP group (p=0.0061). Readmission rates within 30 days were similar between the groups, with 3.41% in the UTAP group and 1.74% in the LTAP group (p=0.4099). Emergency room visits within 30 days were also comparable, with 6.82% in the UTAP group and 7.56% in the LTAP group (p=0.8283) (Table 2).

In comparing UTAP and LTAP with no TAP, several key outcomes were observed (Tables 3, 4). For primary outcomes, the median total amount of MME within the first 24 h was significantly lower in both the UTAP and LTAP groups compared to the no TAP group, with UTAP at 63 MME and LTAP at 98.8 MME, vs. 120 MME in the no TAP group (p<0.0001 for both). On the second day, the UTAP group's median MME was 30, which showed no significant difference from the no TAP group's 30 MME (p=0.3152). However, the LTAP group had a significantly lower median MME of 23 compared to 30 in the no TAP group (p=0.0231). Over the entire hospital stay, the UTAP group had a significantly lower total MME of 93 compared to 149.5 in the no TAP group (p<0.0001), while the LTAP group had 120 MME compared to 149.5 in the no TAP group (p<0.0001).

**Table 3:** Primary and secondary outcomes comparison between patients who received UTAP blocks and those who did not receive TAP in postoperative pain management. Primary outcomes include the total amount of MME consumed within the first 24 h, on the second postoperative day, and during the entire hospital stay. Secondary outcomes include total anesthesia time, pain scores (based on pain scale ranging from 0 {no pain} to 10 {worst possible pain}, used to assess patients' subjective pain levels) at various intervals (postoperative day 1, entering the PACU, 1 h into PACU, and at discharge), LOS, and rates of 30-day readmissions and ER visits. Data are presented as medians with IQR, means with SD, or percentages as appropriate. The p-values indicate the statistical significance of differences between the UTAP and no TAP groups (p<0.05). UTAP: ultrasound-guided transversus abdominis plane block; LTAP: laparoscopic-guided transversus abdominis plane block; MME: morphine milligram equivalents; PACU: postanesthesia care unit; SD: standard deviation; IQR: interquartile range; LOS: length of stay; ER: emergency room

Primary outcomes	Ultrasound TAP		No TAP		p-Value
	Median	Interquartile range	Median	Interquartile range	
Total amount of milligrams of morphine equivalents (MME) within the first 24 h	63	30-95	120	92.5-153	<0.0001
Total amount of milligrams of morphine equivalents (MME) second day	30	15-45	30	15-45	0.3152
Total amount of milligrams of morphine equivalents (MME) during the entire stay	93	45-140	149.5	109.5-192.5	<0.0001
Secondary outcomes					
Total anesthesia time	98.5	86-117	86	77-98	<0.0001
	Pain score mean (SD)	Pain score range	Pain score mean (SD)	Pain score range	
Pain score entering PACU	2.48 (3.7)	0-10	2.35 (3.4)	0-10	0.9237
Pain score 1 h into PACU	5.31 (3.4)	0-10	5.55 (2.8)	0-10	0.5235
Pain score postoperative day 1	4.07 (2.5)	0-10	4.59 (2.5)	0-10	0.0635
Pain score at discharge	3.94 (2.7)	0-10	4.24 (2.4)	0-10	0.2692
Length of stay	Mean (SD)	Range	Mean (SD)	Range	0.0228
	1.59 (1.3)	1-10	1.28 (0.7)	0-8	
30-day readmission	Number	Percentage	Number	Percentage	0.4175
	3	3.41%	19	1.94%	
30-day emergency room visit	6	6.82%	81	8.27%	0.6345

**Table 4:** Primary and secondary outcomes comparison between patients who received LTAP blocks and those who did not receive TAP in postoperative pain management. Primary outcomes include the total amount of MME consumed within the first 24 h, on the second postoperative day, and during the entire hospital stay. Secondary outcomes include total anesthesia time, postoperative pain scores (based on pain scale ranging from 0 {no pain} to 10 {worst possible pain}, used to assess patients' subjective pain levels) at various intervals (postoperative day 1, entering the PACU, 1 h into PACU, and at discharge), LOS, and rates of 30-day readmissions and ER visits. Data are presented as medians with IQR, means with SD, or percentages, as appropriate. The p-values indicate the statistical significance of differences between the LTAP and no TAP groups (p<0.05). UTAP: ultrasound-guided transversus abdominis plane block; LTAP: laparoscopic-guided transversus abdominis plane block; MME: morphine milligram equivalents; PACU: postanesthesia care unit; SD: standard deviation; IQR: interquartile range; LOS: length of stay; ER: emergency room

Primary outcomes	Laparoscopic TAP		No TAP		p-Value
	Median	Interquartile range	Median	Interquartile range	
Total amount of milligrams of morphine equivalents (MME) within the first 24 h	98.8	75-130	120	92.5-153	<0.0001
Total amount of milligrams of morphine equivalents (MME) 2nd day	23	15-45	30	15-45	0.0231
Total amount of milligrams of morphine equivalents (MME) during entire stay	120	84-165	149.5	109.5-192.5	<0.0001
Secondary outcomes					
Total anesthesia time	92.5	83-104.5	86	77-98	<0.0001
	Pain score mean (SD)	Pain score range	Pain score mean (SD)	Pain score range	
Pain score entering PACU	2.35 (3.4)	0-10	2.52 (3.5)	0-10	0.5620
Pain score 1 h into PACU	5.44 (2.5)	0-10	5.55 (2.8)	0-10	0.6313
Pain score postoperative day 1	4.52 (2.7)	0-10	4.59 (2.5)	0-10	0.7685
Pain score at discharge	4.05 (2.1)	0-9	4.24 (2.4)	0-10	0.3337
	Mean (SD)	Range	Mean (SD)	Range	
Length of stay	1.18 (0.8)	0-5	1.28 (0.7)	0-8	0.1400
	Number	Percentage (%)	Number	Percentage (%)	
30-day readmission	3	1.74%	19	1.94%	1.0000
30-day emergency room visit	13	7.56%	81	8.27%	0.7547

The median total anesthesia time was longer in both the UTAP (98.5 min) and LTAP (92.5 min) groups compared to the no TAP group (86 min) (p<0.0001 for both). Pain scores on postoperative day 1 were slightly lower in the UTAP group (4.07) compared to the no TAP group (4.59), though this difference was not statistically significant (p=0.0635). The LTAP group had a similar pain score (4.52) compared to the no TAP group (4.59) (p=0.7685). Pain scores upon entering PACU were similar across all groups: UTAP at 2.48, LTAP at 2.35, and no TAP at 2.52 (p=0.9237 for UTAP and p=0.5620 for LTAP). One hour into PACU, pain scores remained similar: UTAP at 5.31, LTAP at 5.44, and no TAP at 5.55 (p= 0.5235 for UTAP and p=0.6313 for LTAP). At discharge, the UTAP group's mean pain score was 3.94, the LTAP group's was 4.05, and the no TAP group's was 4.24 (p=0.2692 for UTAP and p=0.3337 for LTAP) (Tables 3, 4).

The LOS was significantly longer for the UTAP group compared to the no TAP group (1.59 days vs. 1.28 days, p=0.0228). The LTAP group had a slightly shorter stay (1.18 days) compared to no TAP, though this difference was not significant (p=0.1400). Readmission and emergency room visit rates within 30 days showed no significant differences among the groups. UTAP had readmission and ER visit rates of 3.41% and 6.82%, respectively; LTAP had rates of 1.74% and 7.56%; and no TAP had rates of 1.94% and 8.27% (Tables 3, 4).

Multiple linear regression analysis identified several predictors for MME use within the first 24 h among groups (Table 5). Age was consistently associated with reduced MME across all comparisons, with significant negative coefficients for UTAP vs. no TAP (p<0.0001) and LTAP vs. no TAP (p<0.0001). Gender shows variability, being non-significant for all groups except LTAP vs. No TAP group (p=0.0219). The ASA rating significantly increases MME for UTAP vs. LTAP (p=0.0036) but is non-significant in other comparisons. Both UTAP and LTAP usage significantly reduce MME across all comparisons (p<0.0001). Pain scores the morning after surgery (p=0.0495 for UTAP vs. LTAP, p<0.0001 for the others), upon entering the PACU (p=0.3724 for UTAP vs. LTAP, p<0.0001 for the others), and 1 h into the PACU (p=0.0279 for UTAP vs. LTAP, p<0.0001 for the others) are significant predictors of increased MME, especially in the UTAP vs. no TAP and LTAP vs. no TAP groups. These findings highlight the influence of age, ASA rating, type of TAP block, and postoperative pain scores on MME usage within the first 24 h postsurgery.

**Table 5:** Predictors for MME within the first 24 h using multiple linear regression analysis. The table shows the results of multiple linear regression analysis assessing predictors of MME consumption within the first 24 h across three comparisons as follows: UTAP vs. LTAP, UTAP vs. no TAP, and LTAP vs. no TAP. Predictors include patient age, gender (male), ASA rating (higher values indicate more severe systemic disease), use of UTAP or LTAP, postoperative pain scores (morning after surgery, entering the PACU, and 1 h into PACU). Regression coefficients represent the magnitude and direction of the predictor's effect on MME consumption, while p-values indicate statistical significance. UTAP: ultrasound-guided transversus abdominis plane block; LTAP: laparoscopic-guided transversus abdominis plane block; MME: morphine milligram equivalents; PACU: postanesthesia care unit; SD: standard deviation; IQR: interquartile range; LOS: length of stay; ER: emergency room; ASA: American Society of Anesthesiologists

Variables	UTAP vs. LTAP		UTAP vs. no TAP		LTAP vs. no TAP	
Predictors	Regression coefficient	p-Value	Regression coefficient	p-Value	Regression coefficient	p-Value
Age (years)	-0.33	0.1722	-0.55	<0.0001	-0.47	<0.0001
Gender (male)	-3.68	0.5734	5.40	0.0776	6.95	0.0219
ASA rating (severe systemic disease)	18.93	0.0036	5.75	0.0573	5.20	0.0854
UTAP or LTAP use	-29.70	<0.0001	-44.44	<0.0001	-15.16	<0.0001
Pain score morning after surgery	2.04	0.0495	3.04	<0.0001	2.56	<0.0001
Pain score entering PACU	0.71	0.3724	1.68	<0.0001	1.46	<0.0001
Pain score 1 h into PACU	2.13	0.0279	2.50	<0.0001	2.28	<0.0001

## Discussion

LSG has rapidly become the most common weight loss procedure in North America, accounting for 152,866 (58.14%) bariatric surgeries in 2021, significantly up from 17.8% in 2011 [15]. This shift in global preference for LSG over laparoscopic Roux-en-Y gastric bypass (LRYGB) has prompted the continuous release of new data, including long-term outcomes. Enhanced Recovery After Surgery (ERAS) protocols, initially implemented for colorectal surgeries, have since been expanded to include most major surgical procedures [16]. With the adoption of ERAS protocols and the trend toward minimizing perioperative narcotic use, various multimodal pain control strategies have been developed.

Bariatric surgery patients are generally restricted from using non-steroidal anti-inflammatory drugs (NSAIDs) due to the risk of gastric and marginal ulcers, which eliminates a common non-opioid medication for pain control. Expanding multimodal pain control options, including the routine use of TAP blocks, can be highly beneficial for this patient population. We elected to retrospectively review patients who had received TAP blocks vs. those who did not and compare outcomes of two methods of administration as follows: UTAP and LTAP.

As documented in previous studies, both UTAP and LTAP are associated with reduced narcotic use [10,11]. However, current data on which method offers better pain control are mixed. Zaghiyan et al. reported that LTAP was superior to UTAP in achieving pain control and minimizing opioid use in colorectal surgery patients [14]. Contrarily, our data suggest the opposite - both UTAP and LTAP significantly reduced pain compared to the control group that received no TAP, but UTAP resulted in significantly lower narcotic use over the hospital stay than LTAP (93 MME vs. 120 MME, p=0.0004). 

We believe that the accuracy of ultrasound guidance improves the accuracy of anesthetic placement within the appropriate interfascial plane, which may explain the better outcomes with UTAP. Our data support this hypothesis, showing significantly lower median MME within the first 24 h in the UTAP group compared to the LTAP group (63 MME vs. 98.8 MME, p<0.0001). The variability in TAP block placement by surgeons without direct visualization of hydrodissection might contribute to the less effective pain control observed with LTAP.

Although the UTAP group had significantly lower opioid consumption, the lack of significant differences in pain scores between the UTAP, LTAP, and no TAP groups could be attributed to the subjective nature of pain assessments and variability in how patients report pain. Pain scores alone may not fully capture the benefits of reduced opioid use, which is associated with fewer opioid-related complications, such as nausea, respiratory depression, and impaired gastrointestinal function. Future studies might benefit from including additional metrics, such as patient satisfaction and quality of recovery, to better evaluate the clinical impact of reduced opioid consumption.

However, it is important to acknowledge that while TAP blocks provide effective somatic analgesia by targeting the abdominal wall nerves (T6-L1), they do not address visceral pain, which originates from the manipulation of internal organs during surgery [9]. In laparoscopic sleeve gastrectomy, visceral pain may persist despite the use of TAP blocks, which could explain the continued need for opioids in some patients. This limitation may contribute to the variations in postoperative opioid use and recovery outcomes observed across the groups. Multimodal pain management strategies, therefore, should consider additional interventions to fully address both somatic and visceral pain.

An interesting observation from our study is the difference in length of stay (LOS) among the groups. The UTAP group had a significantly longer LOS compared to the LTAP group (1.59 days vs. 1.18 days, p=0.0061). This finding is contrary to our expectations and previous studies which have shown reduced LOS with TAP blocks [12]. The increased LOS in the UTAP group might be attributed to a higher age of patients in this group (mean age 57.7 years) and ASA classification compared to the LTAP and no TAP groups, as older and less healthy patients often have slower recovery times and more postoperative complications [17]. Older patients and those with greater comorbidities often require longer recovery times, independent of the quality of pain control. This may have overshadowed the potential benefits of reduced opioid use in this group. Future prospective studies should stratify patients based on demographic and clinical factors to better understand their influence on recovery outcomes.

Furthermore, while pain scores were not significantly different among the groups entering the PACU and at discharge, there was a trend towards lower pain scores in the UTAP group on postoperative day one (mean 4.07) compared to the no TAP group (mean 4.59, p=0.0635). Although not statistically significant, this trend suggests a potential benefit of UTAP in early postoperative pain control, which warrants further investigation.

Additionally, the multiple linear regression analysis identified key predictors of MME use within the first 24 h postsurgery. Across all comparisons, older age was associated with a reduction in MME consumption and was significant in the UTAP vs. no TAP group and LTAP vs. no TAP. Male gender was a significant predictor of higher MME use in the LTAP vs. no TAP group. These findings suggest the need to tailor pain management strategies based on patient demographics. A higher ASA rating, indicative of more severe systemic disease, was associated with increased MME use in the UTAP vs. LTAP group. This highlights the importance of considering patient comorbidities when planning postoperative analgesia.

Regression analysis also showed the use of UTAP or LTAP blocks significantly reduced MME consumption compared to no TAP across all comparisons, with the most pronounced effect seen in the UTAP vs. no TAP group. This reinforces the importance of UTAP blocks for precise placement and efficacy. Finally, postoperative pain scores were significant predictors of MME consumption, particularly in patients who did not receive TAP blocks. Higher pain scores the morning after surgery and 1 hour into PACU were associated with increased opioid use. Pain scores entering the PACU showed a weaker association with MME use than scores measured later, such as the morning after surgery or 1 hour into the PACU. This likely reflects the residual effects of intraoperative anesthesia and sedation, which may blunt initial pain perception immediately postsurgery. The relationship between pain scores and MME use was less pronounced in the UTAP vs. LTAP comparison, likely reflecting the superior analgesic coverage provided by both techniques. These findings underscore the importance of addressing early postoperative pain to minimize opioid requirements and associated complications. The enhanced precision of UTAP appears to provide more effective pain relief, reinforcing its role in multimodal analgesia protocols for bariatric surgery.

This study has several limitations, primarily related to its retrospective design, which inherently lacks the standardization and randomization seen in prospective trials. One notable limitation is the variability in the choice of local anesthetic between the UTAP and LTAP groups. In the UTAP group, 0.5% ropivacaine was used, while the LTAP group received 0.25% bupivacaine. Although bupivacaine is considered more potent than ropivacaine, and we might expect it to produce a more profound sensory block at a lower concentration, our results showed that ultrasound-guided TAP blocks with ropivacaine had a more profound effect in lowering postoperative opioid consumption.

This variability in local anesthetic protocols introduces potential differences in block efficacy, but the observed outcomes suggest that, in this case, the ultrasound guidance technique may play a more significant role in block effectiveness than the choice of anesthetic itself. Despite the theoretical advantage of bupivacaine in providing a stronger block, the precision of ultrasound-guided placement in the UTAP group likely contributed to the superior results observed, as demonstrated by the significant reduction in opioid use compared to the LTAP group.

Additionally, there may have been variability among the surgeons in how TAP blocks were performed, especially for the LTAP group, as laparoscopic-guided blocks are more dependent on the surgeon's technique. This lack of standardization could have introduced variability in the outcomes. Another limitation is the retrospective nature of this study, which makes it prone to selection bias. Patient selection may have been influenced by surgeon preference, anesthesiologist input, or patient-specific factors that were not fully accounted for in the analysis.

Future prospective, randomized trials are necessary to eliminate these variables and further validate our findings. Such studies should standardize the protocols for both UTAP and LTAP to ensure consistency in anesthetic combinations and techniques. Despite these limitations, our data suggest that UTAP may be superior to LTAP due to the increased accuracy in targeting the nerves within the lateral abdominal wall musculature.

## Conclusions

In conclusion, our study highlights the potential benefits of UTAP over LTAP in reducing postoperative opioid consumption and improving pain control in patients undergoing LSG. These findings support the integration of UTAP blocks into multimodal pain management protocols for bariatric surgery patients. Further research is needed to optimize anesthetic techniques and combinations for enhanced patient outcomes.
